# Commentary: High Intensity Physical Rehabilitation Later Than 24 h Post Stroke Is Beneficial in Patients: A Pilot Randomized Controlled Trial (RCT) Study in Mild to Moderate Ischemic Stroke

**DOI:** 10.3389/fneur.2020.00182

**Published:** 2020-03-10

**Authors:** Mari Nakao, Masahiro Banno, Yuki Kataoka, Shunsuke Taito

**Affiliations:** ^1^Rehabilitation Department, Niigata Rehabilitation Hospital, Niigata, Japan; ^2^Rehabilitation Department, Tohoku University Graduate School of Medicine, Sendai, Japan; ^3^Division of Dysphagia Rehabilitation, Niigata University Graduate School of Medical and Dental Science, Niigata, Japan; ^4^Department of Psychiatry, Seichiryo Hospital, Nagoya, Japan; ^5^Department of Psychiatry, Nagoya University Graduate School of Medicine, Nagoya, Japan; ^6^Systematic Review Workshop Peer Support Group (SRWS-PSG), Japan; ^7^Hospital Care Research Unit, Hyogo Prefectural Amagasaki General Medical Center, Amagasaki, Japan; ^8^Department of Healthcare Epidemiology, Graduate School of Medicine and Public Health, Kyoto University, Kyoto, Japan; ^9^Department of Respiratory Medicine, Hyogo Prefectural Amagasaki General Medical Center, Amagasaki, Japan; ^10^Division of Rehabilitation, Department of Clinical Practice and Support, Hiroshima University Hospital, Hiroshima, Japan

**Keywords:** rehabilitation, stroke, bias, intensity, modified Rankin Scale

## Introduction

This article is a general commentary on “High Intensity Physical Rehabilitation Later Than 24 h Post Stroke is Beneficial in Patients: A pilot Randomized Controlled Trial (RCT) Study in Mild to Moderate Ischemic Stroke (1). A recent, current Cochrane review (2) concluded that very early intervention is not beneficial for patients, although the adequate dose of rehabilitation is unknown. Tong et al. (1) reported that early intensive mobilization after stroke was beneficial compared with very early mobilization and early routine mobilization. This finding is important for clinicians who prescribe rehabilitation for acute stroke patients. However, we believe that several concerns should be noted regarding the methods in Tong's study.

## Methods

To focus on potential biases in the present study, we evaluated the risk of bias (3). In a recent, current Cochrane review entitled “Very early vs. delayed mobilization after stroke” (2), nine studies were selected as eligible for meta-analysis, and the risk of bias was evaluated. We adopted the same method as this Cochrane review.

## Results

From the evaluation, we found several points that could affect the results of the present study (Table 1).

**Table 1:** Risk of bias (3).

**Item**	**Author's judgment**	**Support for judgment**
Selective reporting (selective reporting bias)	High	Comment: The primary outcome of the manuscript (1), the modified Rankin Scale, was not listed in the study protocol (4). This unreported primary outcome measurement was used for sample size calculation (1). The outcomes registered in the study protocol (4) were the NIHSS, Barthel Index, Rivermead Motor Assessment, Fugl-Meyer and MRI brain scans. There were no reports on the outcomes of these variables in the manuscript (1).
Blinding of outcome assessor (detection bias)	Unclear	Comment: There was no information or the assessor of the outcome was blinded.
Incomplete outcome data (attribution bias)	High	Comment: The authors removed participants who did not complete the intervention from each assigned group (lack of intervention fidelity).
Blinding (performance bias)	High	Comment: Therapists who performed the intervention could not be blinded.
Random sequence generation (selection bias)	Low	Quote: “All patients were randomly assigned to three groups by a computer generated randomization procedure.”
Allocation concealment? (selection bias)	Low	Quote: “using opaque envelopes.”

*(1), (3), and (4) indicate references*.

### Outcome Variables and the Assessment of Outcomes

There was no report on the outcome of variables registered in the protocol (4) in the manuscript (1), and this causes a high selective reporting bias. The primary outcome defined in the manuscript (1) was a modified Rankin Scale (mRS) score, which was not listed in the protocol (4). The outcomes described in the study protocol (4) included the NIHSS, Barthel Index, Rivermead Motor Assessment, Fugl-Meyer Assessment, and MRI brain scans. The authors should clearly report the outcomes to be assessed in the trial protocol (4) and should also describe the measurement methods (1) to reduce biases.

The authors explained in the manuscript (1) that they estimated the sample size from the prevalence of the primary outcome (mRS score [0–2]) at 3 months after stroke onset. The process of determining the sample size for the trial protocol (4) was not mentioned. The sample size calculation and outcome variables should be determined before registration to reduce the risk of bias.

Additionally, no information on whether the outcome assessor was blinded was provided in the manuscript (1) or the protocol (4). This can result in unclear detection bias. The authors should state whether the assessor was blinded in the outcome assessment section (1).

### Method of Intervention

The unclear intervention period for each patient causes a high risk of performance bias. The intervention periods ranged from 10 to 14 days depending on cases. The intervention period for each patient and the method of determining this period were not described in the protocol (4) or the manuscript (1). Moreover, blinding of this type of intervention is impossible because of its nature. If therapists were not blinded, the intervention period in the Early Intensive Mobilization (EIM) group could have been several days longer than that in the other two groups, which could cause a high risk of performance bias. Therefore, the authors should clarify the median and quartile of the intervention period for each group.

### Lack of Information on Patients During the Observation Period and an Unknown Accumulative Dose Effect of Rehabilitation Before Outcome Measurement

Without controlling the accumulative dose of rehabilitation during the observation period, it is difficult to evaluate whether early or very early intervention were effective. We assumed that the accumulative dose of rehabilitation from the endpoint of the intervention to the time of outcome measurement had a greater effect on the outcome than the timing of the intervention. In the manuscript (1), no information was provided about patients during the observation period. The length of hospital stay, the number of patients transferred to inpatient rehabilitation, and the accumulative volume of rehabilitation before evaluation at 3 months could affect the outcome. According to a previous study (5) a higher rehabilitation dose results in a better outcome. Another previous study (6) reported the accumulative dose effect on stroke patients during the recovery period (2 to 6 months after stroke onset). The authors should describe this information as the AVERT Trial Collaboration group did in their report (7, 8).

### Lack of Intervention Fidelity

The study (1) has a possible high risk of attribution bias because of the policy regarding participant dropout and a lack of adherence to the intervention.

In the Early Routine Mobilization (ERM) group 16 patients dropped out because of “excessive mobilization.” In the Very Early Intensive Mobilization (VEIM) and EIM groups, 13 and 10 patients, dropped out because they could not achieve mobilization. Tong et al. (1) removed these participants from the analysis because they did not complete the intervention that was intended for their group. This can cause high risk of attribution bias, which can change the outcomes. To correct this bias, the authors should undertake an intention-to-treat analysis that includes all randomized participants in the intervention groups to which they were randomized, regardless of the intervention that they actually received. “Drop outs” should only refer to participants who actively chose to withdraw from the study or who were lost to follow-up for other reasons.

## Discussion

From the risk of bias evaluation (3), we determined that the conclusion of the manuscript (1) was vulnerable, and more information is needed to show the clear usefulness of high-dose (intensive) early intervention after stroke. High risks of selective reporting, attribution and performance bias (Table 1) could distort the study design described in the protocol (4) and affect the interpretation of the results. The number of high-risk biases in the study by Tong et al. (1) surpassed those of the nine studies included in a recent, current Cochrane review (2). A considerable difference between the study protocol (4) and the manuscript (1) was observed for some outcome variables. The accumulative dose during the observational period for each patient group needs to be considered, and detailed information about rehabilitation exposure during the recovery period should be disclosed.

## Author Contributions

MN wrote a draft of the manuscript. MN, MB, YK, and ST contributed to the manuscript revision and read and approved the submitted version.

### Conflict of Interest

The authors declare that the research was conducted in the absence of any commercial or financial relationships that could be construed as a potential conflict of interest.
